# Identification and Characterization of Critical Processing Parameters in the Fabrication of Double-Emulsion Poly(lactic-co-glycolic) Acid Microparticles

**DOI:** 10.3390/pharmaceutics16060796

**Published:** 2024-06-12

**Authors:** Elizabeth R. Bentley, Stacia Subick, Michael Pezzillo, Stephen C. Balmert, Aidan Herbert, Steven R. Little

**Affiliations:** 1 Department of Bioengineering, University of Pittsburgh, 302 Benedum Hall, 3700 O’Hara Street, Pittsburgh, PA 15260, USA; erb122@pitt.edu; 2 Department of Chemical Engineering, University of Pittsburgh, 940 Benedum Hall, 3700 O’Hara Street, Pittsburgh, PA 15213, USA; sas567@pitt.edu (S.S.); mjp179@pitt.edu (M.P.); 3 Department of Dermatology, University of Pittsburgh School of Medicine, W1150 Biomedical Science Tower, 200 Lothrop Street, Pittsburgh, PA 15213, USA; scb22@pitt.edu; 4 DigiM Solution—Pixel Perfect Therapeutics, 500 W Cummings Park, Suite 3650, Woburn, MA 01801, USA; aidan.herbert@digimsolution.com; 5 Department of Clinical and Translational Science, University of Pittsburgh, Forbes Tower, Suite 7057, Pittsburgh, PA 15213, USA; 6 McGowan Institute for Regenerative Medicine, University of Pittsburgh, 450 Technology Drive, Suite 300, Pittsburgh, PA 15219, USA; 7 Department of Immunology, University of Pittsburgh, 200 Lothrop Street, Pittsburgh, PA 15213, USA; 8 Department of Pharmaceutical Sciences, University of Pittsburgh, 3501 Terrace Street, Pittsburgh, PA 15213, USA; 9 Department of Ophthalmology, University of Pittsburgh, 203 Lothrop Street, Pittsburgh, PA 15213, USA

**Keywords:** design of experiments, quality by design, microparticles, double emulsion, drug delivery, controlled release, polymers, critical processing parameters, critical quality attributes

## Abstract

In the past several decades, polymeric microparticles (MPs) have emerged as viable solutions to address the limitations of standard pharmaceuticals and their corresponding delivery methods. While there are many preclinical studies that utilize polymeric MPs as a delivery vehicle, there are limited FDA-approved products. One potential barrier to the clinical translation of these technologies is a lack of understanding with regard to the manufacturing process, hindering batch scale-up. To address this knowledge gap, we sought to first identify critical processing parameters in the manufacturing process of blank (no therapeutic drug) and protein-loaded double-emulsion poly(lactic-co-glycolic) acid MPs through a quality by design approach. We then utilized the design of experiments as a tool to systematically investigate the impact of these parameters on critical quality attributes (e.g., size, surface morphology, release kinetics, inner occlusion size, etc.) of blank and protein-loaded MPs. Our results elucidate that some of the most significant CPPs impacting many CQAs of double-emulsion MPs are those within the primary or single-emulsion process (e.g., inner aqueous phase volume, solvent volume, etc.) and their interactions. Furthermore, our results indicate that microparticle internal structure (e.g., inner occlusion size, interconnectivity, etc.) can heavily influence protein release kinetics from double-emulsion MPs, suggesting it is a crucial CQA to understand. Altogether, this study identifies several important considerations in the manufacturing and characterization of double-emulsion MPs, potentially enhancing their translation.

## 1. Introduction

Drug delivery systems can be characterized as technologies that enable the improved biodistribution and retention, controlled release, and/or increased specificity of active pharmaceutical ingredients (e.g., proteins, small molecule drugs, nucleic acids, etc.) [1,2]. In the past few decades, a variety of drug delivery system formats have emerged, including hydrogels, nanoparticles, microparticles, liposomes, and micelles. For the local delivery of therapeutic proteins and/or drugs, polymeric microparticles are readily used due to their significant advantages. For instance, if engineered to be >10 μm in diameter, microparticles can avoid phagocytosis and clearance through the reticuloendothelial system, facilitating sustained placement duration in vivo [3,4]. In addition, microparticles have a small surface area-to-volume ratio when compared to other delivery systems (e.g., nanoparticles), leading to potentially slower (and more extended) controlled drug release [4]. Therefore, if parenterally administered, it may be possible to achieve drug delivery over weeks to months using microparticle-based drug delivery systems, as opposed to hours and/or days, a frequent limitation of other delivery approaches.

Polymeric microparticles can be formulated using a variety of approaches, including but not limited to emulsification/solvent evaporation, spray drying, electrospraying, and microfluidics [4,5,6,7]. Of the methods for microparticle fabrication, perhaps the most used and well-established method is emulsification/solvent evaporation. In this process, cargo is encapsulated in either single or multiple emulsions [5,7,8]. Single-emulsion techniques are commonly used to encapsulate lipophilic compounds, as these compounds are normally soluble in the same organic solvent as the polymer and less prone to degradation [5,8]. Hydrophilic compounds (e.g., proteins), however, are more often encapsulated using a double-emulsion process to provide hydrophilic pockets for the cargo within the polymeric core, enabling protection from degradation [5,7,8,9].

**Figure 1:**
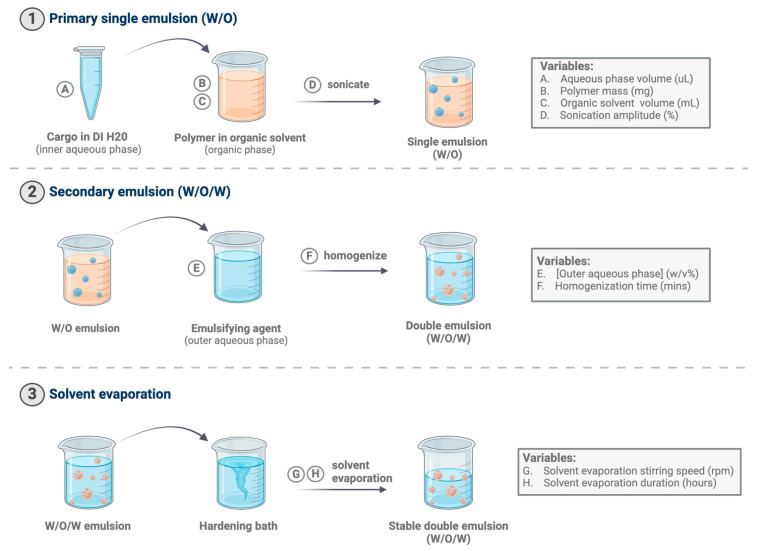
Process diagram illustrating emulsion process and potential critical processing parameters in double-emulsion solvent evaporation microparticle fabrication. Critical processing parameters can exist in all steps of fabrication, including the primary emulsion (W/O), secondary emulsion (W/O/W), and solvent evaporation steps.

In comparison to double-emulsion processes, single-emulsion manufacturing processes are much simpler and more streamlined. More specifically, single-emulsion microparticles are formulated by first adding either water-insoluble active ingredients (e.g., soluble in chloroform or dichloromethane) or, alternatively, a solid suspension of water-soluble active ingredients (e.g., proteins), to an organic solvent containing polymer. The resulting mixture is then added to an aqueous phase containing an emulsifying agent that stabilizes the emulsion and is sonicated or homogenized to form the single emulsion. The solvent then diffuses from the droplets and evaporates, leaving solid microparticles. Double-emulsion microparticles, in contrast, are formulated similarly, but with additional steps (Figure 1, Steps 1–3). In this instance, water-soluble cargo is first dissolved in what will ultimately become an inner aqueous phase. The inner aqueous phase can also include additional excipients (e.g., Tween, bovine serum albumin (BSA), etc.) to stabilize the cargo and protect it from potential degradation [10]. The inner aqueous phase, containing protein and excipients, is then added to an immiscible organic phase containing polymer in an organic solvent (Figure 1, Step 1). This two-phase mixture is then sonicated, forming the primary emulsion (W/O). After the primary emulsion is formed, it is then added to an additional aqueous phase containing an emulsifying agent and homogenized, forming the secondary emulsion (water-in-oil-in-water, W/O/W) (Figure 1, Step 2). Emulsification is followed by solvent evaporation and polymer precipitation, resulting in polymeric microparticles that encapsulate the desired cargo (Figure 1, Step 3).

Additional steps during fabrication can increase process requirements during manufacturing, potentially limiting the translation of these technologies [3,11]. To date, very few (~20) injectable poly (lactic-co-glycolic acid) (PLGA)-based delivery systems are FDA-approved despite their extensive use in basic science and preclinical research [12,13]. Of the microparticle-based FDA-approved delivery systems, most deliver small molecules (e.g., dexamethasone, triamcinolone acetonide, etc.) and/or peptides (e.g., leuprolide, octreotide, etc.), allowing for fabrication using single-emulsion versus double-emulsion methods [13]. Therefore, there is a crucial need to increase the translatability of double-emulsion systems. To do so, it is increasingly important for there to be a better understanding of the manufacturing processes of these delivery systems. Without such an understanding of the double-emulsion microparticle manufacturing parameters and process(es), the ability to successfully scale technologies to a larger manufacturing scale is not as accessible [14].

To address limitations and knowledge gaps pertaining to the manufacturing of double-emulsion microparticles, researchers can utilize a quality by design (QbD) approach for pharmaceutical development. As described by the International Conference of Harmonization, QbD is a systematic approach to drug development with the goals of increasing process capability, increasing manufacturing efficiencies, and reducing product variability [14,15,16]. Using QbD, researchers can develop a quality target product profile (QTPP) for delivery systems [14,15,16]. The QTPP of a delivery system is a summary of its key product characteristics, otherwise known as critical quality attributes (CQAs) [14,15,17]. CQAs are greatly impacted by critical material attributes (CMAs) and critical processing parameters (CPPs) used in a manufacturing process. In the context of polymeric delivery systems, CMAs are properties or characteristics of raw input materials, such as polymer composition and molecular weight [14,15,18]. CPPs, on the other hand, are manipulable variables during manufacturing processes [17]. For polymeric microparticles manufactured via emulsification evaporation, CPPs can include emulsification time and stirring rate, to name a few [15]. Altogether, the identification of CMAs and CPPs, as well as their relationships to CQAs, informs the QTPP of a product. Overall, a well-defined QTPP assists greatly with product translation.

To develop a QTPP and enhance the translatability of double-emulsion microparticles, this study sought to identify and characterize significant CPPs and CQAs in blank (no therapeutic protein) and protein-loaded double-emulsion microparticle fabrication. To identify CPPs, a design of experiments (DOE) approach can be used. DOE is a statistical tool that allows researchers to manipulate multiple parameters at a time, rather than one at a time, to determine their impact on outcome variables [16,17,19]. With this approach, researchers can reduce the amount of material and time required to conduct a thorough investigation of the desired parameters [17,19]. While there are many types of DOEs, a response surface methodology design (RSM) allows researchers to explore single-factor effects, interaction effects, and quadratic effects of input parameters [16,19,20]. In many cases, the relationship between an input CPP and outcome CQA is linear. The inclusion of quadratic effects in this model enables researchers to identify curvature in a response [16]. For these reasons, this study first uses RSM as a tool to identify CPPs that have a significant impact on double-emulsion microparticle size. Size was initially chosen as the primary CQA in this study due to the well-established relationship between delivery vehicle size and release kinetics [4,9].

Following the identification of CPPs, their impact on the CQAs of protein-loaded microparticles (specifically recombinant human CCL22 microparticles (rhCCL22-MPs)) was then studied in an effort to increase the process capability and manufacturing efficiency of a batch-based manufacturing process, two objectives of QbD. CCL22 is a chemokine for regulatory T cells, a population of immunosuppressive T cells found naturally within the body [21]. Local delivery of CCL22-MPs has been shown to suppress inflammation in both small and large animal models of inflammation (e.g., periodontitis, transplant rejection, etc.) [22,23,24]. To aid in the translation of rhCCL22-MPs, the impact of CPPs on CQAs such as size, surface morphology, and protein release kinetics was evaluated. Through the novel application of a machine learning algorithm, the impact of CPPs on inner occlusion size and interconnectivity, two CQAs less commonly analyzed, were also quantitatively assessed. Historically, there are challenges with quantitatively assessing microparticle internal structure (e.g., inner occlusion size, etc.) and overall porosity. Porosity is often measured via porosimetry and gas adsorption techniques, but these approaches are irreversible and often require large amounts of samples (e.g., >200 mgs) [25]. Furthermore, approaches such as porosimetry are rarely used to study closed pores, such as those in double-emulsion microparticles [26]. Therefore, many researchers rely upon the visual and qualitative examination of microparticle internal structure and porosity using scanning electron microscopy (SEM) images. The use of a machine learning algorithm to quantitatively assess attributes of microparticle internal structure addresses many of these limitations. Altogether, with this information and the data provided by the QbD analysis, a scaled formulation of rhCCL22-MPs, which uses a larger polymer input but maintains significant CQAs, was identified, increasing the overall process capability and efficiency of this manufacturing process.

## 2. Materials and Methods

### 2.1. Materials

Poly(lactic-co-glycolic) acid (PLGA; RG502H, 50:50 lactic/glycolic acid, MW: 7000–17,000 kDa) was supplied by Sigma-Aldrich (St. Louis, MO, USA). Bovine serum albumin (BSA) was purchased from R&D Systems (Minneapolis, MN, USA). Polyvinyl alcohol (PVA; MW ~25%, 98% hydrolyzed) was obtained from Polysciences (Warrington, PA, USA). Sodium chloride (NaCl) was purchased from Fisher Scientific (Waltham, MA, USA). Recombinant human CCL22 (rhCCL22; 69 a.a.) was obtained from PeproTech (Cranbury, NJ, USA). Enzyme-linked immunosorbent assays (ELISAs; rhCCL22) were obtained from R&D Systems (Minneapolis, MN, USA).

### 2.2. Experimental Design

The experimental design was generated using Design-Expert^®^ Stat-Ease (version 13, Stat-Ease, Inc., Minneapolis, MN, USA). A response surface methodology (RSM) with a two-level, split-plot design was used to allow for hard-to-change variables (based on academic laboratory constraints) to be incorporated into the design space. In addition, a D-optimal design was used to focus on identifying significant processing parameters. The resulting design included 9 discrete or continuous independent variables (CPPs): (1) aqueous phase volume (continuous), (2) polymer amount (continuous), (3) organic solvent volume (continuous), (4) sonication amplitude (discrete), (5) outer aqueous phase concentration (continuous), (6) homogenization time (continuous), (7) solvent evaporation speed (discrete), and (8) solvent evaporation duration (continuous). These parameters, as well as their levels, are summarized in Table 1. Based on the generated design, 60 different blank (no therapeutic protein) double-emulsion microparticle batches were formulated. The order of experiments was randomized, and blocks were added to distribute batches over a five-day period, with a random number of batches formulated each day.

After formulation, Design-Expert^®^ Stat-Ease (version 13, Stat-Ease, Inc., Minneapolis, MN, USA) was used to evaluate the effect of CPPs on microparticle size, the main response variable in the initial study. Data did not exhibit a normal distribution; therefore, a natural log transformation was performed before further analysis (Figure S1). An analysis of variance (ANOVA) was used to determine significant factors. Auto-select with AICc-Backwards was used to remove insignificant model terms that may be repetitive or that could lead to poorly estimated model coefficients. To maintain a hierarchy, some insignificant single-effect variables were reintroduced to the model and are displayed in the ANOVA results (e.g., sonication amplitude, solvent evaporation duration, etc.).

### 2.3. Fabrication of PLGA Microparticles

#### 2.3.1. Blank MPs

Blank PLGA microparticles (containing no therapeutic protein) were formulated using a previously published double-emulsion solvent evaporation method (W/O/W), with modifications made for the parameters mentioned in Section 3.2 [22]. The inner aqueous phase contained 10 mg/mL of BSA and 15 mM of NaCl in DI H_2_0_._ For the organic phase, PLGA was dissolved in dichloromethane (DCM). To form the water-in-oil emulsion, the inner aqueous phase was added to the organic phase (PLGA in DCM) and briefly sonicated at a specified amplitude for 10 s. The resulting emulsion was then poured into 60 mL of DI H_2_0 containing PVA and homogenized at 3000 rpm for a specified amount of time. The resulting water-in-oil-in-water emulsion was then poured into 80 mL of DI H_2_0 containing PVA located on a stir plate at room temperature. The mixture was stirred at a set RPM for a specific amount of time to allow for DCM to evaporate. Following initial fabrication steps, the resulting microparticles were washed four times with deionized water to remove residual PVA. The washed particles were then lyophilized for 72 h and stored at −20 °C until further analysis.

#### 2.3.2. CCL22 MPs

CCL22 MPs were formulated using the double-emulsion solvent evaporation method (W/O/W) described above. In addition to containing 10 mg/mL of BSA and 15 mM of NaCl, the inner aqueous phase was loaded with 25 ug of recombinant human CCL22 (rhCCL22). BSA and NaCl concentrations in the inner aqueous phase were maintained throughout all studies.

### 2.4. Characterization of PLGA Microparticles

#### 2.4.1. Blank MPs

The main CQAs of blank microparticles include microparticle size and surface morphology. For the initial DOE, the CQA was microparticle size. To determine microparticle diameters, microparticles were sized using volume impedance measurements (Beckman Coulter Counter, Brea, CA, USA; *n* = 10,000 particles). Additionally, surface morphology was assessed using scanning electron microscopy (SEM, Zeiss, Jena, Germany) after sputter coating (Denton Sputter Coater).

#### 2.4.2. CCL22-MPs

For protein-loaded microparticles, additional CQAs include inner occlusion size, inner occlusion interconnectivity, protein release kinetics, and protein loading capacity. To assess the inner microsphere morphology, cross-sectional scanning electron microscopy images were acquired at 1.50 kx magnification (*n* = 3 per batch). Utilizing a random forest machine learning classifier through “digiM I2S”, a cloud pharmaceutical image processing and data management platform (DigiM Solution, LLC, Woburn, MA, USA), the occlusions and polymeric regions could be identified. Random forest image segmentation relies on an array of decision trees dividing pixels into pre-defined categories, based on textural and spatial relationships with neighboring pixels. The classifiers were trained using a supervised learning process, in which the analyst provided direct feedback during training to influence the labeling performance. Once the occlusions and polymeric regions have been identified, their area, size, and shape can be quantified. The size of the inner occlusions can be determined by converting pixel dimensions to real-world units determined using the imaging device (SEM). A watershed algorithm is used to differentiate neighboring occlusion regions before computing the size and shape of each identified occlusion and polymeric region. To assess inner occlusion interconnectivity, the percent polymer (% polymer) of a cross-section was calculated. More specifically, the area of the polymer matrix was first divided by the total area of the cross-section. These values were provided by the “digiM I2S” analysis described above. The resulting fraction was then multiplied by 100 to get a percent (% polymer of cross-section). To assess rhCCL22 release kinetics, ~5 mg of MPs (*n* = 3 per batch) was suspended in 1 mL of PBS with 1% BSA and incubated at 37 °C with end-over-end rotation. Suspended MPs were sampled periodically following centrifugation, allowing for supernatants to be extracted. rhCCL22 concentration in supernatants was then quantified using enzyme-linked immunosorbent assays (ELISAs). Release curves, demonstrating cumulative release over time (mass protein released/mass of particles), were then plotted. To plot rhCCL22 release as a percent of encapsulated, the loading capacity of formulations was determined. To assess loading capacity, ~5 mg of MPs (*n* = 3 per batch) was dissolved in dichloromethane and rhCCL22 was extracted three times into 0.25 mL volumes of PBS + 0.1% sodium dodecyl sulfate (SDS). rhCCL22 concentration in extracted samples was then determined via ELISA. Loading capacity (ng rhCCL22/mg MP) was then determined by dividing the amount of rhCCL22 encapsulated (ng rhCCL22) by the initial microparticle amount (mg of MPs) in each sample.

## 3. Results and Discussion

### 3.1. Analysis of Design of Experiments Model

For initial studies, microparticle size was chosen as the main CQA because there is a known correlation between size and release kinetics. In addition, there are application-based considerations in microparticle fabrication. Small microparticles (<10 um) can be phagocytosed via the reticuloendothelial system, leading to clearance [9,27,28]. Large microparticles can potentially spark a foreign body response [27,28]. For this application (local chemokine delivery), it is important to manufacture microparticles that will be maintained at the site of interest, creating a local depot, while avoiding phagocytosis and an adverse immune reaction. Therefore, it is important to first study how these critical processing parameters impact microparticle size.

Blank microparticle batches were first formulated using the CPPs generated through the RSM design. Following formulation, microparticles were sized, and log-transformed mean particle diameters (volume%) were input into Design-Expert^®^ Stat-Ease (Stat-Ease, Inc., Minneapolis, MN, USA) with a quadratic model, enabling the identification of single-factor effects, interaction effects, and quadratic effects. Using the quadratic model, a low standard deviation was obtained (0.2189). In this instance, the standard deviation refers to the amount of random variation left in the process after accounting for model effects, indicating the model can describe the size response with accuracy. For this model, a high R^2^ value (0.8773) was obtained. The adjusted R^2^ value, which is the amount of variation in the data explained by the model, is also high (0.8306). Since these values are close to 1, it can be inferred that the regression model fits the input data well [20].

### 3.2. Identification of Critical Processing Parameters in Double-Emulsion Microparticle Fabrication

Several single-factor and interaction effects were found to have a significant impact on blank microparticle size (Table 2). Significant linear single-factor effects include aqueous phase volume, solvent volume, PLGA amount, homogenization time, and the concentration of surfactant (PVA) in the outer aqueous phase. For interaction effects, the interactions between aqueous phase volume and solvent volume, solvent volume and the amount of PLGA, homogenization time and solvent evaporation duration, and outer aqueous phase surfactant concentration and solvent evaporation duration were found to be significant. The model also found a significant quadratic effect of solvent volume on size.

### 3.3. Effect of Single-Factor and Interaction Variables on Unloaded Double-Emulsion Microparticle Size

#### 3.3.1. Single-Factor Effects

**Figure 2:**
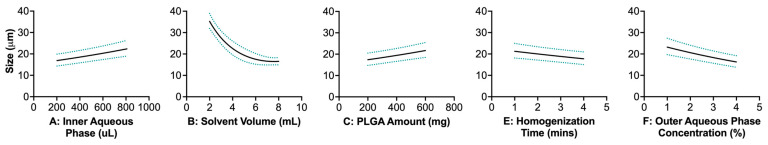
Single-factor plots demonstrating the significant relationship between microparticle size and significant critical processing parameters (*p* < 0.05), including (Parameter A) inner aqueous phase volume (*n* = 200), (Parameter B) solvent volume (*n* = 200), (Parameter C) PLGA amount (*n* = 200), (Parameter E) homogenization time (*n* = 200), and (Parameter F) concentration of surfactant in the outer aqueous phase (*n* = 200). The black solid line represents the expected trend, and the blue dotted line illustrates the confidence bands for that trend, as predicted by the RSM.

As mentioned previously, several single-factor CPPs were identified in double-emulsion microparticle fabrication. According to the RSM, the relationship between most CPPs and microparticle size (mean microparticle diameter (μm)) is linear (Figure 2). However, there is a quadratic relationship between solvent volume and microparticle size (Figure 2). Trends for insignificant parameters can be found in the Supplementary Materials (Figure S2).

##### Inner Aqueous Phase Volume

Microparticle size significantly increased as aqueous phase volume increased (Figure 2). More specifically, the size increased from 16.9 to 22.4 μm as the aqueous phase volume increased from 200 to 800 μL. Since water (inner aqueous phase) has a greater viscosity than the solvent (DCM) in this instance (H_2_O @ 25 °C = 0.89 cP; DCM @ 25 °C = 0.41), it is possible that increasing inner aqueous phase volume increases the overall viscosity of the dispersed phase. With an increased viscosity, more shear force or energy is required to generate smaller particles [9,29,30]. Thus, larger inner aqueous phase volumes may result in larger microparticles.

##### Solvent Volume

Solvent volume has a significant quadratic effect on microparticle size. As solvent volume increased, microparticle size decreased (Figure 2). Microparticle size decreased quadratically from 35.2 to 16.6 μm as DCM volume increased from 2 to 8 mL. Changes in solvent volume can significantly impact the viscosity of the organic phase. As discussed previously, more viscous mixtures require more shear force or energy to generate smaller particles [9,29,30]. Therefore, at a set energy level, larger solvent volumes, resulting in a lower polymer concentration and/or viscosity, may result in smaller microparticles [9,11,30,31]. It is important to note that the effect is not linear; once a certain solvent volume threshold is reached, there is less of a reduction in microparticle size (Figure 2). There is a known non-linear relationship between viscosity and polymer concentration [32,33,34]. As polymer concentration increases, or solvent volume decreases at a set polymer amount, a threshold is reached, after which viscosity increases significantly [32,33]. In this study, the threshold appears to be around 4 mL of solvent. Below this threshold, there is a significant increase in microparticle size, potentially due to the relationship between viscosity, polymer concentration, and microparticle size.

##### Amount of Poly(lactic-co-glycolic) Acid

The amount of PLGA also significantly impacted microparticle size (Figure 2). As the amount of PLGA increased from 200 to 600 mg, microparticle size increased from 17.4 to 21.7 μm. Similar to solvent volume, changing the amount of PLGA in the organic phase influences polymer concentration. In turn, increased polymer concentration results in increased organic phase viscosity, resulting in larger microparticles for reasons discussed above [9,11,29,30,31].

##### Homogenization Time

Homogenization time was also found to have a significant impact on microparticle size (Figure 2). As homogenization time increased from 1 to 4 min, microparticle size decreased linearly from 21.3 μm to 17.8 μm. In this scenario, increasing homogenization time may impact the polydispersity index (PDI) of the resulting microparticles, leading to a decrease in mean diameter. Although not statistically significant, increased homogenization times appear to decrease microparticle polydispersity (PDI), a metric used to describe how much size variation exists (Figure S3). It is possible that increased homogenization times result in sustained energy input that can break up larger particles that were contributing to larger polydispersity.

##### Outer Aqueous Phase Concentration

The concentration of PVA (*w*/*v* %) within the outer aqueous phase significantly decreased microparticle size as concentration increased (Figure 2, Table 2). Increased concentrations of emulsifier can reduce droplet surface tension, collisions, and coalescence, enabling smaller droplet formation and stabilization [9,29,31,35,36]. However, it is important to note that excessive PVA concentrations can result in aggregation [9,31]. Therefore, it is possible that the trend identified within this range (1% to 4%) is not the same outside of this range. As a result, there may be a need to be aware of this phenomenon in new formulations.

#### 3.3.2. Interaction Effects

While four interaction effects had a statistically significant impact on microparticle size, two interaction effects (AB and BC) more significantly impacted microparticle size and will be the focus of subsequent sections. The other two interaction effects did not impact microparticle size as drastically (Figure S4).

##### Inner Aqueous Phase Volume and Solvent Volume

**Figure 3:**
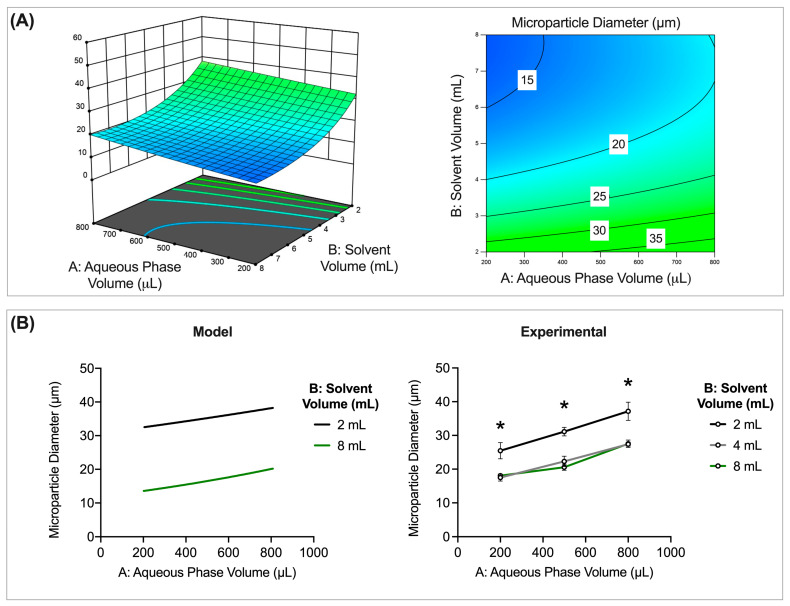
Aqueous phase volume (μL) and solvent volume (mL) interact to impact microparticle size. (**A**) A 3D surface (left) and contour plot (right) illustrating the overall trends of the impact of aqueous phase volume and solvent volume on microparticle size. (**B**) Model and experimental data examining size trends at specific levels of aqueous phase volume and solvent volume. Other preparation conditions for these batches are as follows: 200 mg of polymer, 3000 rpm homogenization speed, 1 min homogenization time, 600 rpm stirring speed, 55% sonication amplitude, 2% outer aqueous phase concentration, and 3 h solvent evaporation. * Indicates significant difference in microparticle size (*p* < 0.05).

The interaction between inner aqueous phase volume (μL) and solvent volume (mL) was found to have a significant impact on microparticle size (*p* = 0.0479). The smallest microparticles can be obtained at low inner aqueous phase volumes (μL) and high solvent volumes (mL) (Figure 3A). Larger microparticles, on the other hand, can be obtained at higher aqueous phase volumes (μL) and solvent volumes (mL) (Figure 3A).

Examining specific levels of inner aqueous phase volume (200, 400, and 600 μL) and solvent volume (2, 4, and 8 mL) further illustrates the relationship between inner aqueous phase volume, solvent volume, and microparticle size. More specifically, smaller microparticles are obtained when an 8 mL solvent is used (Figure 3B). As discussed previously, size differences as a result of changes in solvent volume can be attributed to modulating polymer concentration. A decrease in polymer concentration reduces organic phase viscosity, resulting in smaller microparticles [9,11,29,30,31]. However, the addition of a third experimental solvent volume level (4 mL) illustrates a notable trend (Figure 3B). More specifically, there is no statistically significant difference in size for microparticles made with either 4 or 8 mL of solvent at each inner aqueous phase volume. There is, however, a statistically significant difference in size for batches formulated with 2 mL and 4 or 8 mL. Therefore, these data suggest that below a certain threshold (<50 mg of polymer/mL of solvent), further reducing polymer concentration may not have an impact on microparticle size. On the contrary, concentrations above this threshold (>50 mg of polymer/mL of solvent) may significantly impact microparticle size. These findings are in accordance with those discussed in Section 3.3.1 (Inner Aqueous Phase Volume), where the quadratic relationship between viscosity and polymer concentration dictates size.

##### Solvent Volume and Polymer Amount

**Figure 4:**
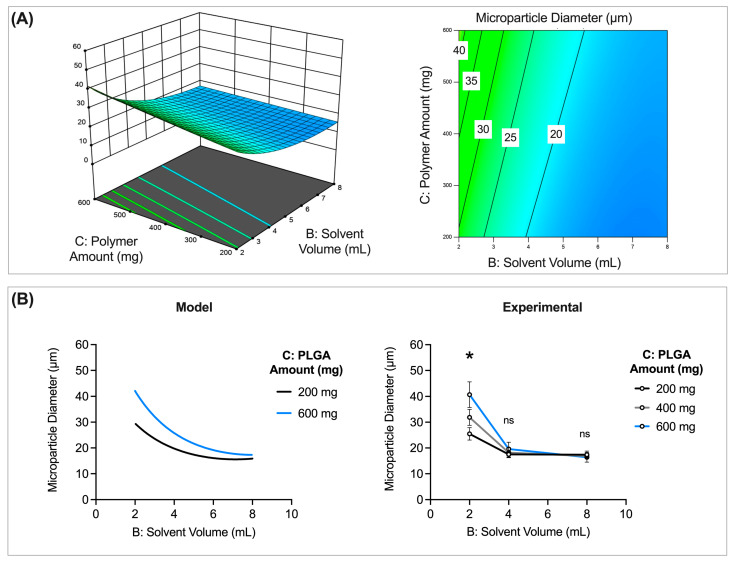
Solvent volume (mL) and polymer amount (mg) interact to impact microparticle size. (**A**) A 3D surface (**left**) and contour plot (**right**) illustrating overall trends of the impact of polymer and solvent volume on microparticle size. (**B**) Model and experimental data examining size trends at specific levels of polymer amount and solvent volume. Other preparation conditions for these batches are as follows: 200 μL inner aqueous phase volume, 3000 rpm homogenization speed, 1 min homogenization time, 600 rpm stirring speed, 55% sonication amplitude, 2% outer aqueous phase concentration, and 3 h solvent evaporation. * Indicates significant difference in microparticle size (*p* < 0.05). ns indicates non-significant difference in microparticle size.

Solvent volume (mL) and polymer amount (mg) used during formulation were found to have an interaction effect that results in a significant difference in microparticle size (*p* = 0.0326). The largest microparticles are obtained for batches formulated with low solvent volumes (mL) but high amounts of polymer (mg) (Figure 4A). Smaller microparticles, on the other hand, can be obtained when high solvent volumes are used, irrespective of the amount of polymer used (Figure 4A).

Assessing specific levels of polymer amount (200, 400, and 600 mg) and solvent volume (2, 4, and 8 mL) further illustrates the relationship between solvent volume, polymer amount, and microparticle size (Figure 4B). The model data suggest that the amount of polymer used in fabrication has a significant impact on microparticle size at lower solvent volumes but less so at higher solvent volumes (Figure 4A,B). The experimental data, with the addition of a third level, confirm these trends (Figure 4B). There is a statistically significant difference in microparticle size for batches made with different polymer amounts when 2 mL of solvent is used (*p* < 0.05). However, at higher solvent volumes (4 or 8 mL), the amount of polymer used in fabrication does not significantly impact microparticle size. These results suggest that increasing the polymer amount at higher solvent volumes may not impact microparticle size. However, at lower solvent volumes, increasing the polymer amount does significantly impact size. Together, the results suggest it may be important to study solvent volume and polymer amount independently for their impacts on CQAs, rather than together as polymer concentration, which is more commonly studied. Furthermore, these results also highlight the importance of studying the interaction of CPPs across multiple levels, as trends may not be the same at each level.

### 3.4. Effect of Critical Processing Parameters on CQAs of rhCCL22-Loaded Microparticles

After identifying and characterizing the effect of CPPs on blank (or unloaded) microparticle size, their impact on the CQAs (e.g., size, surface morphology, inner occlusion size, interconnectivity, etc.) of recombinant human CCL22-loaded microparticles (rhCCL22-MPs) was studied to enhance the translatability of this technology.

#### 3.4.1. Interaction between Inner Aqueous Phase Volume and Solvent Volume

To study the interaction between inner aqueous phase volume and solvent volume, batches of microparticles were manufactured using three different levels per variable (inner aqueous phase volume: 200 μL, 500 μL, and 800 μL; solvent volume: 2 mL, 4 mL, and 8 mL). In these experiments, the polymer amount remained constant based on the published effective formulation (200 mg) [22].

**Figure 5:**
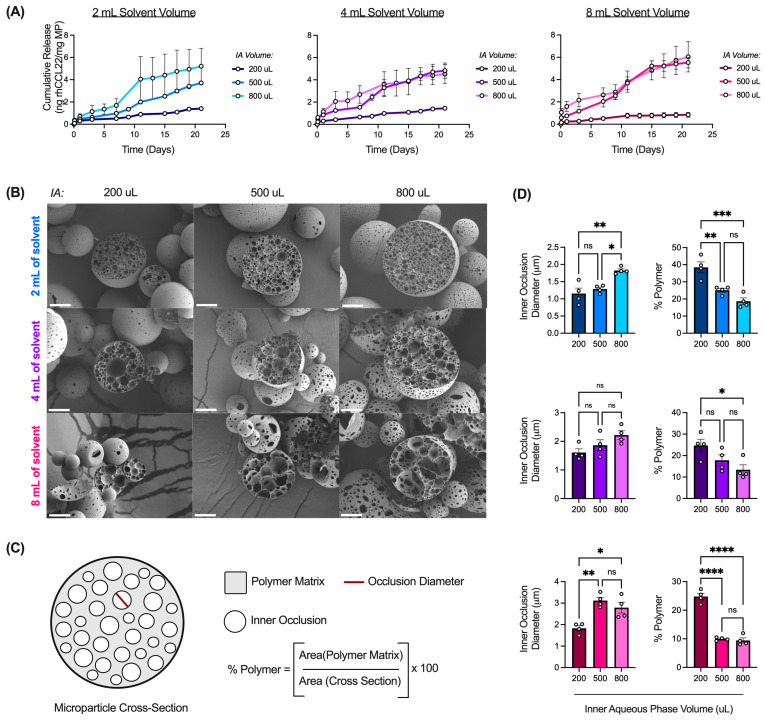
Interaction between inner aqueous phase volume and solvent volume impacts release and internal structure of rhCCL22-MPs. (**A**) Cumulative release (ng rhCCL22/mg MP) profiles of rhCCL22-MPs formulated with each inner aqueous phase volume (200, 500, and 800 μL) at each solvent volume (2, 4, and 8 mL) (*n* = 3). (**B**) Scanning electron microscopy (SEM) images of microparticle cross-sections. SEM images taken at 1.5 kx. Scale bar = 10 μm. (**C**) Diagram depicting features of microparticle internal structure and associated measurements. (**D**) Microparticle inner occlusion diameter (μm) and polymer matrix composition (% polymer) measurements (*n* = 3–4). Other preparation conditions for these batches are as follows: 200 mg of PLGA, 3000 rpm homogenization speed, 1 min homogenization time, 55% sonication amplitude, 600 rpm stirring speed, 2% outer aqueous phase concentration, and 3 h solvent evaporation. * *p* < 0.05, ** *p* < 0.01, *** *p* < 0.001, **** *p* < 0.0001, ns indicates non-significant difference.

Notably, the interaction between the inner aqueous phase volume and solvent volume modulates rhCCL22 release kinetics (Figure 5A). Overall, increasing inner aqueous phase volume from 200 μL to 500 μL or 800 μL accelerated rhCCL22 release across all solvent volumes. These differences in release kinetics may be explained by several variables, including microparticle size, surface morphology, and internal structure. In some instances, larger microparticles can promote slower release kinetics due to a smaller surface area-to-volume ratio [4,9]. However, in this case, although increasing inner aqueous phase volume increased microparticle size, release kinetics were accelerated, rather than suppressed (Figure S5 and Figure 5A). Therefore, rhCCL22 release may be controlled by other factors, such as microparticle surface morphology. For instance, surface porosity has previously been shown to impact CCL22 release, as porous microparticles exhibited increased release when compared to non-porous microparticles [24]. In the current study, as the inner aqueous phase volume increases, the surface porosity increases (Figure S6). It is likely that larger inner aqueous phase volumes promote the loss of the internal aqueous phase towards the outer aqueous phase during solvent evaporation, resulting in increased porosity and subsequently, increased protein release [6,11,37].

In addition to microparticle surface morphology, microparticle internal structure may also modulate rhCCL22 release kinetics. More specifically, it is possible that changes in inner aqueous phase volume can impact inner occlusion diameter and interconnectivity, or the space between occlusions, potentially changing the % polymer of a microparticle cross-section (Figure 5B). As the inner aqueous phase volume increases, the inner occlusion diameter appears to increase (Figure 5C,D). Greater inner aqueous phase volumes are associated with increased droplet coalescence as a result of shorter inter-droplet spacing [11]. Furthermore, as the inner occlusion diameter increases, it approaches the diameter of the overall microparticle, resulting in a thinner polymeric film between droplets [30]. Droplet coalescence can then further increase, as the thin polymer layer is unable to resist Laplace pressures, or the pressure difference between the inside and outside of a curved surface, during solvent evaporation and microparticle drying [6,30]. Taken together, it is likely that an increasing inner aqueous phase volume increases the overall droplet coalescence, resulting in an increased inner occlusion diameter and thinner polymeric layers between droplets (Figure 5D). As a result, inner occlusion interconnectivity, or the polymer between inner occlusions (% polymer), decreases (Figure 5D).

Given that release is often controlled by water penetration and subsequent polymer degradation, the reduced space of the polymer barrier between inner occlusions could enhance rhCCL22 release, as the polymer degrades, and inner occlusions connect to release protein. This is consistent with prior studies that suggest that the coalescence of inner occlusions to enable release is especially important for larger biomolecules, such as proteins, which cannot otherwise pass through the polymer matrix [38,39]. Furthermore, it has been shown that the release of positively charged proteins may be impeded by negatively charged matrices due to protein–polymer interactions [15,40,41]. rhCCL22 is a positively charged chemokine, and the polymer used in these studies, carboxylic acid terminated PLGA, is negatively charged. Therefore, it is likely that increasing inner aqueous phase volume from 200 μL to 500 or 800 μL results in increased droplet coalescence, inner occlusion diameter, and inner occlusion interconnectivity, resulting in increased release kinetics (Figure 5A). Notably, there are limited differences in inner occlusion interconnectivity or % polymer for batches made with 500 and 800 μL inner aqueous phase volumes (Figure 5D). Accordingly, these batches demonstrate similar release profiles (Figure 5A). It is possible that the factors controlling droplet coalescence (e.g., Laplace pressures) are similar for 500 and 800 μL inner aqueous phase volumes but significantly different when compared to 200 μL. As a result, batches made with 500 or 800 μL inner aqueous phase volumes share similar features in regard to microparticle internal structure (e.g., inner occlusion size, interconnectivity), resulting in similar release kinetics.

#### 3.4.2. Interaction between Solvent Volume and Polymer Amount

To study the interaction between solvent volume and polymer amount, batches of microparticles were formulated using three different levels per variable (solvent volume: 2 mL, 4 mL, and 8 mL; polymer amount: 200 mg, 400 mg, and 600 mg). In these experiments, the inner aqueous phase volume remained constant (200 μL).

Overall, microparticle size is significantly impacted by increasing polymer amounts at 2 mL of solvent, but not at 4 or 8 mL of solvent (Figure S7). It is likely that increasing the polymer amount at 2 mL of solvent significantly increases the viscosity of the dispersed phase, resulting in increased microparticle size [9,29,30,31]. In contrast, there is no significant difference in microparticle size between batches made with 200, 400, or 600 mg at 4 or 8 mL of solvent. Altogether, sizing results are in accordance with those discussed in Section 3.3.2. (Solvent Volume and Polymer Amount) for blank microparticles and highlight the importance of studying multiple levels of critical processing parameters, as well as their interactions, as trends may not be the same across levels.

**Figure 6:**
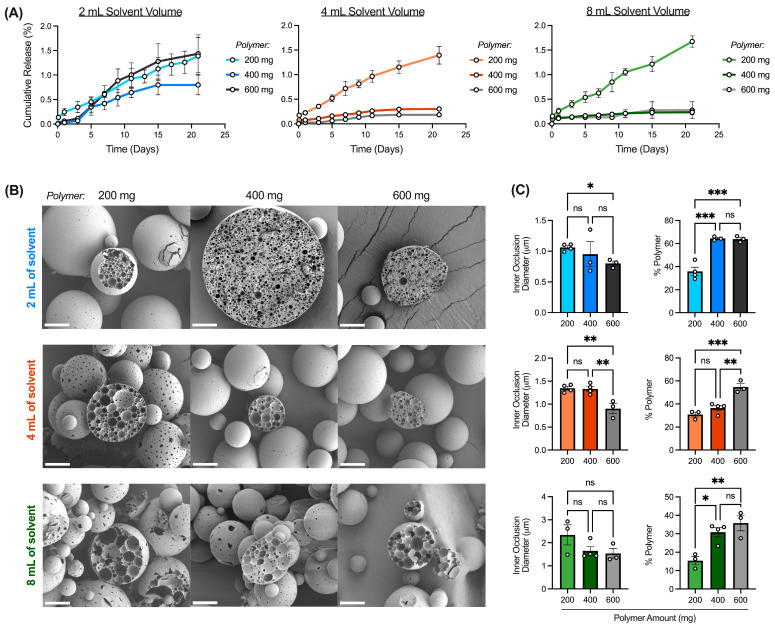
Interaction between polymer amount and solvent volume impacts release kinetics and internal structure of rhCCL22-MPs. (**A**) Cumulative release (%) profiles of rhCCL22-MPs formulated with each polymer amount (200, 400, and 600 mg) at each solvent volume (2, 4, and 8 mL) (*n* = 3). (**B**) Scanning electron microscopy (SEM) images of microparticle cross-sections. SEM images taken at 1.5 kx. Scale bar = 10 μm. (**C**) Microparticle inner occlusion diameter (μm) and polymer matrix composition (% polymer) measurements (*n* = 3–4). Other preparation conditions for these batches are as follows: 200 μL IA phase volume, 3000 rpm homogenization speed, 1 min homogenization time, 55% sonication amplitude, 600 rpm stirring speed, 2% outer aqueous phase concentration, and 3 h solvent evaporation. * *p* < 0.05, ** *p* < 0.01, *** *p* < 0.001, ns indicates non-significant difference.

In addition to microparticle size, the interaction between polymer amount and solvent volume results in differences in rhCCL22 release kinetics (Figure 6A). To assess the impact of different polymer amounts on rhCCL22 release, cumulative release as a percent of protein loaded can be analyzed. In this case, cumulative rhCCL22 release represents the amount of protein released (ng rhCCL22/mg MP) over the experimental loading capacity (ng rhCCL22/mg MP). Displaying rhCCL22 release as a percent of loaded rhCCL22 eliminates different experimental loading capacities as an explanation for differences in release kinetics (Figure S8). In this study, there are limited differences in rhCCL22 release for batches made with different polymer amounts when 2 mL of solvent is used, despite significant differences in microparticle size (Figure 6A and Figure S7). However, at 4 or 8 mL of solvent, rhCCL22 release is repressed when the polymer amount is increased to 400 or 600 mg, despite similar size distributions (Figure 6A and Figure S7). There is little to no difference in rhCCL22 release for batches made with 400 or 600 mg at these solvent volumes (Figure 6A). These results support the notion that rhCCL22 release is not predominantly controlled by microparticle size.

As discussed previously, modulating critical processing parameters, such as solvent volume and polymer amount in this case, may impact other critical quality attributes (e.g., surface morphology, inner occlusion size, and interconnectivity), leading to differences in release kinetics. In this scenario, factors that change because of increased polymer amounts (e.g., inner occlusion size, inner occlusion interconnectivity) may govern rhCCL22 release. Increasing the polymer amount from 200 mg to 400 or 600 mg appears to reduce inner occlusion diameter (D_i_) (Figure 6B,C). As a result, D_i_ is much smaller than the overall microparticle diameter (D_m_) (D_i_ << D_m_), preventing droplet coalescence, increasing emulsion stability, and increasing the polymer matrix thickness as a barrier between the pores (Figure 6C) [30]. Accordingly, increasing the polymer amount from 200 mg to 400 or 600 mg results in a matrix with a greater composition of polymer (% polymer) at each solvent volume (Figure 6C). As discussed previously, with smaller pores and increased % polymer between pores, cumulative rhCCL22 release is repressed when 400 or 600 mg of polymer is used at higher solvent volumes (4 or 8 mL) (Figure 6A). However, when 2 mL of solvent is used, there are limited differences in release kinetics despite differences in inner occlusion diameter and polymer composition (Figure 6A). As mentioned previously, when the polymer amount is increased when 2 mL of solvent is used, microparticle size increases significantly (Figure S7). It is possible that a significant increase in size can counterbalance repressed release caused by increased % polymer due to increased autocatalysis and acidification associated with larger matrices [37,39].

### 3.5. Applying Principles of DOE to Scale-Up Batch Manufacturing

In the studies above, the interactions between (1) inner aqueous phase volume and solvent volume as well as (2) solvent volume and polymer amount were explored. In these studies, increasing inner aqueous phase volume accelerated rhCCL22 release, whereas increasing polymer amount suppressed rhCCL22 release, respectively. Based on these results, it was hypothesized that increasing the inner aqueous phase volume while increasing the polymer amount may yield a formulation with similar CQAs (e.g., release kinetics) to the previously published formulation, despite scaling to a larger polymer input [22]. Thus, the interaction between the inner aqueous phase volume and polymer amount was studied to identify a potential new formulation with a larger polymer input.

#### 3.5.1. Interaction between Inner Aqueous Phase Volume and Polymer Amount

To assess the interaction between the inner aqueous phase volume and polymer amount, 18 batches of microparticles were made. Since scaling polymer amount in an effort to increase batch size was the primary interest, and batches with 400 and 600 mg exhibited increased release kinetics at 2 mL when compared to 4 or 8 mL (Figure 6), 2 mL of solvent volume was chosen for the first nine batches. The remaining batches were made with 4 mL of solvent to serve as a reference point, as the original published formulation utilizes 4 mL [22]. All three inner aqueous phase levels were tested (200 μL, 500 μL, and 800 μL). All other levels of critical processing parameters (e.g., sonication amplitude, homogenization speed, solvent evaporation duration, etc.) were kept constant and in accordance with the previously published formulation [22].

**Figure 7:**
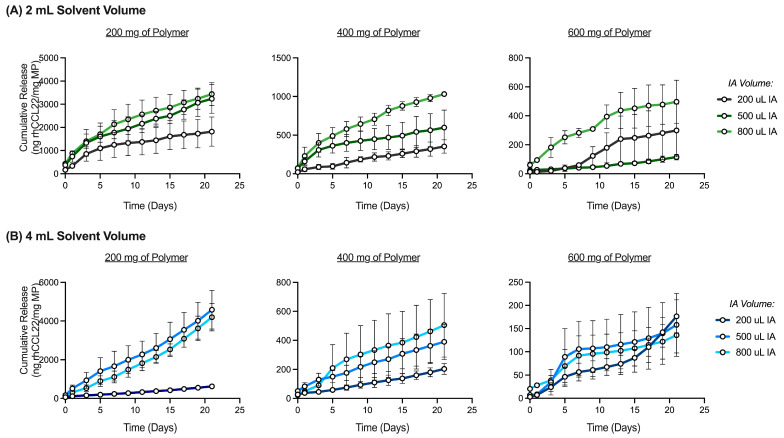
Interaction between solvent volume and polymer amount impacts rhCCL22 release kinetics. Cumulative rhCCL22 release (ng rhCCL22/mg MP) from microparticles formulated with (**A**) 2 mL and (**B**) 4 mL of solvent. Other preparation conditions for these batches are as follows: 3000 rpm homogenization speed, 1 min homogenization time, 55% sonication amplitude, 600 rpm stirring speed, 2% outer aqueous phase concentration, and 3 h solvent evaporation.

When 2 mL of solvent and 200 mg of polymer are used, increasing the inner aqueous phase volume from 200 to 500 or 800 μL accelerated release (Figure 7A). However, at 400 or 600 mg of polymer, there are more significant differences in release depending on the inner aqueous phase volume. At these polymer amounts, batches made with 800 μL inner aqueous phases exhibit greater release kinetics than those formulated with 200 or 500 μL inner aqueous phases. As discussed previously, it is likely that increasing inner aqueous phase volume increases both surface porosity and inner occlusion interconnectivity, resulting in accelerated release. When 4 mL of solvent is used, microparticles made with 500 μL or 800 μL inner aqueous phases behave similarly across polymer amounts (Figure 7B). In addition, these batches demonstrate increased rhCCL22 release when compared to batches made with 200 μL inner aqueous phases. These results are in accordance with prior results, where increased inner aqueous phase volumes (500 or 800 μL) increased pore interconnectivity and inner occlusion size, potentially enhancing rhCCL22 release. Altogether, these results enhance our understanding of the interaction effects between critical processing parameters, potentially enhancing the translation of this technology.

#### 3.5.2. Identifying a Formulation of CCL22-MPs with Increased Batch Size

With a more comprehensive understanding of interaction effects, it may be possible to identify formulations that can increase the process capability and efficiency of this batch-based manufacturing process. One way to increase process capability and efficiency is to identify formulations that use a larger polymer input, increasing the overall batch size. By identifying a formulation with a larger input polymer amount, it may be possible to reduce the number of batches required in a batch-based manufacturing process to achieve a certain quantity (mg) of microparticles. Furthermore, such a formulation would significantly assist in meeting the demands of larger preclinical or clinical studies.

Despite a larger polymer input, it is important to still meet CQAs (e.g., surface morphology, size distribution, rhCCL22 release) of a previously identified, therapeutically effective formulation. More specifically, previously developed CCL22-MPs exhibited spherical morphology with limited porosity, a size distribution between 10–35 μm, and a cumulative release of about 0.50 ng of CCL22/mg of MPs by day 21 [22]. While there are several CQAs of interest to meet, rhCCL22 release kinetics is the primary CQA for this study given that is the most important variable for therapeutic efficacy.

**Figure 8:**
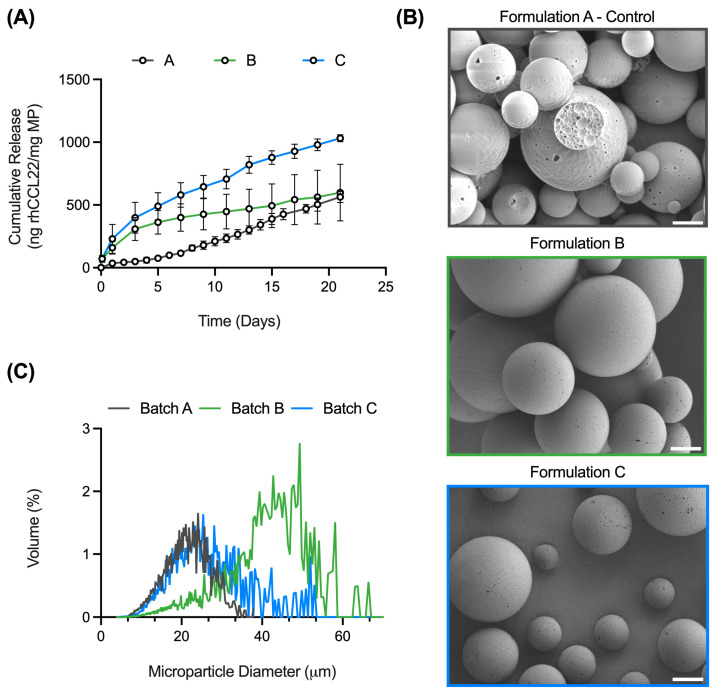
Formulations A, B, and C exhibit some similarities and differences in CQAs. (**A**) Cumulative release curves (ng rhCCL22/mg MP) for formulations A, B, and C. (**B**) Scanning electron microscopy images demonstrating spherical surface morphology. Images were taken at 1.2 kx. Scale bars = 10 μm. (**C**) Volume-weighted size distributions of formulations A, B, and C. *n* = 10,000 particles. Data from formulation A (control) adapted from Fisher et al., *Sci. Adv*. (2019). [22].

To this end, two experimental batches from the above batches meet the release criteria set by previously published works (formulation A) (Table 3). Both formulations were formulated with 2 mL of solvent and 400 mg of polymer but with 500 μL (formulation B) or 800 μL (formulation C) inner aqueous phases (Table 3). These batches exhibit sufficient cumulative rhCCL22 release by day 21, despite differences in several critical processing parameters (e.g., solvent volume, polymer amount, inner aqueous phase volume) (Table 3, Figure 8A). Furthermore, these batches also demonstrate similar spherical surface morphology with limited porosity (Figure 8B). However, there is a significant difference in size between formulations A and B (Figure 8C). Formulations A and C exhibit a similar size distribution (Figure 8C). As previously discussed, reducing solvent volume results in larger microparticles when 200 mg of polymer is used. While formulations B and C were formulated with 400 mg of polymer, they were made with 2 mL of solvent, as opposed to 4 mL of solvent (formulation A), potentially increasing their size. However, it may be possible that increasing the inner aqueous phase volume to 800 μL (formulation C) limits the impact of reduced solvent volume at 400 mg of polymer. To counteract the increase in size for formulation B, homogenization speed, a variable not examined in this study but known to have an effect on microparticle size, may need to be increased [8,18]. Nonetheless, given the similarity in size distribution between formulations A and C, as well as the smaller standard deviation in release for formulation C, formulation C is the most optimal formulation for an increased batch size. Future studies requiring increased microparticle production can utilize the critical processing parameters identified for formulation C.

## 4. Conclusions

Despite being widely researched in an academic setting, there are limited FDA-approved poly(lactic-co-glycolic) acid microparticle-based delivery systems on the market, especially for the sustained release of therapeutic proteins. One potential barrier to the translation of these products is a lack of understanding with regard to critical processing parameters (CPPs) in the manufacturing process of double-emulsion microparticles and their relationship to the critical quality attributes (CQAs) of the resulting microparticles. To address this knowledge gap and add to the translatability knowledge base of double-emulsion microparticles, this study sought to identify and explain CPPs and CQAs in blank and protein-loaded double-emulsion microparticle fabrication. Results from this study indicate that the most significant CPPs impacting many CQAs of double-emulsion microparticles are CPPs within the primary emulsion process (e.g., inner aqueous phase volume, solvent volume, etc.) and their interactions. In addition, it can be concluded that trends in CQAs may not be maintained at different levels of CPPs, suggesting the importance of always studying interaction effects at multiple levels.

A more in-depth exploration of CPPs and CQAs can also assist in explaining release behavior for protein-loaded microparticles, such as rhCCL22-MPs. There are multiple release mechanisms for polymeric microparticles, including diffusion- and erosion/degradation-based release as well as electrostatic release mechanisms [2,40,41,42,43,44,45,46]. Diffusion-based release is more common for small molecules that can readily move through the polymeric matrix with little resistance [38,39,42,45]. Erosion-based release relies upon polymer degradation following matrix hydration, leading to the formation of a pore network that enables the further diffusion of encapsulated agents [2,38,39,42,44,45,47]. Polyester-based delivery systems (e.g., PLGA), such as the rhCCL22-MPs studied here, often exhibit release kinetics characteristic of both diffusion- and erosion-based mechanisms [2,48]. There are many CQAs to consider for these release mechanisms, including microparticle size, surface morphology, and internal structure. From this study, it can be concluded that rhCCL22 release is not predominantly controlled by microparticle size. Furthermore, it can be concluded that while initial diffusion-based release (burst release) may be linked to surface morphology and porosity, long-term rhCCL22 release is more likely controlled by microparticle internal structure [45]. In these studies, an increase in inner occlusion size, resulting in an increase in inner occlusion interconnectivity or a decrease in % polymer of a cross-section, significantly accelerated rhCCL22 release. In this instance, increased inner occlusion interconnectivity could represent an already established percolation network, leading to more rapid coalescence of inner occlusions and the development of pathways for efficient protein diffusion [38]. It is important to note that changes in the % polymer of a cross-section could also alter the magnitude of electrostatic interactions between the polymer and the encapsulated protein. Prior studies have illustrated the importance of considering polymer–drug interactions when explaining release kinetics, as interactions between positively charged cargo (e.g., rhCCL22) and negatively charged matrices (e.g., carboxylic-acid terminated PLGA) can impede release [40,41,43,44]. Therefore, the amount of polymer surface area that the encapsulated agent is in contact with is potentially also a rate-limiting step for electrostatically driven release. Ultimately, both of these mechanisms of release for rhCCL22 support the conclusion drawn in this study that microparticle internal structure is an important CQA. Altogether, these findings illustrate how CPPs in the development and manufacturing of double-emulsion microparticle delivery systems impact their CQAs, potentially increasing the translatability of the technology.

## Figures and Tables

**Table 1:** Manufacturing variables and their corresponding levels used within the design of experiments (DOE) array.

Factors	Name	Units	Minimum	Maximum
A	aqueous phase volume	μL	200	800
B	solvent volume	mL	2	8
C	PLGA amount	mg	200	600
D	sonication amplitude	%	25	100
E	homogenization time	mins	1	4
F	outer aqueous phase concentration	%	1	4
G	solvent evaporation duration	hrs	2	8
H	stirring speed	rpm	100	600

**Table 2:** Analysis of variance (ANOVA) results demonstrating significant single-factor and interaction effects in double-emulsion microparticle fabrication. *p* < 0.05 is deemed significant.

ANOVA Results		*p*-Value
Terms		
Whole plot	D—sonication amplitude	0.8315
Subplot	A—aqueous phase volume	<0.0001
Single-Factor	B—solvent volume	<0.0001
	C—PLGA amount	0.0001
	E—homogenization time	0.0016
	F—outer aqueous phase concentration	<0.0001
	G—solvent evaporation duration	0.7189
Interactions	AB	0.0479
	BC	0.0326
	CD	0.0784
	EG	0.0431
	FG	0.0243
Quadratic	B^2^	0.0069

**Table 3:** Experimental formulations B and C meet release CQA of control formulation A despite differing critical processing parameters.

Formulation	Solvent Volume (mL)	Polymer Amount (mg)	Inner Aqueous Phase (μL)	D21 Cumulative Release (ng rhCCL22/mg MP)	
				Mean	SD
A	4	200	200	0.564	0.043
B	2	400	500	0.598	0.23
C	2	400	800	1.031	0.028

## Data Availability

The raw data supporting the conclusions of this article will be made available by the authors upon request.

## Supplementary Materials

The following supporting information can be downloaded at: https://www.mdpi.com/article/10.3390/pharmaceutics16060796/s1, Figure S1: Non-transformed and log transformed microparticle size distributions from design of experiments study; Figure S2: Single factor plots demonstrating the relationship between microparticle size and insignificant critical processing parameters; Figure S3: Microparticle polydispersity index (PDI) decreases slightly as homogenization time increases; Figure S4: Homogenization time and outer aqueous phase concentration both interact with solvent evaporation duration to impact microparticle size; Figure S5: Increasing inner aqueous phase volume increases size and polydispersity of rhCCL22-MPs; Figure S6: Microparticle surface porosity increases as solvent volume (mL) and inner aqueous phase volume (μL) increase; Figure S7: Increasing polymer amount at 2 mL of solvent volume impacts size and polydispersity of rhCCL22-MPs; Figure S8: Impact of polymer amount and solvent volume on rhCCL22 loading capacity.

## Abbreviations

BSA	Bovine serum albumin
CMAs	Critical material attributes
CPPs	Critical processing parameters
CQAs	Critical quality attributes
DOE	Design of experiments
IA	Inner aqueous phase
MP(s)	Microparticle(s)
NaCl	Sodium chloride
O/W	Oil-in-water (single) emulsion
PLGA	Poly(lactic-co-glycolic acid)
PVA	Polyvinyl alcohol
QbD	Quality by design
QTPP	Quality target product profile
SEM	Scanning electron microscopy
W/O/W	Water–oil–water (double) emulsion
