# Supplementary material for: Identification and Characterization of Critical Processing Parameters in the Fabrication of Double-Emulsion Poly(lactic-co-glycolic) Acid Microparticles
Source: Pharmaceutics. 2024 Jun 12;16(6):796. doi: 10.3390/pharmaceutics16060796 (PMC11207479; doi:10.3390/pharmaceutics16060796)
Supplement: Supplementary file 1 [file pharmaceutics-16-00796-s001.zip › pharmaceutics-3040367-supplementary.pdf]

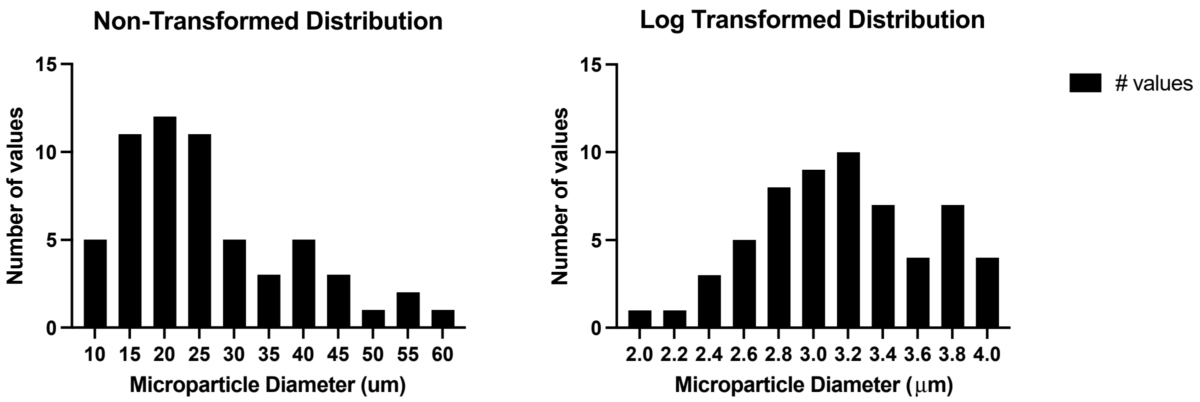

Figure S1. Non-transformed and log transformed microparticle size distributions from design of experiments study.

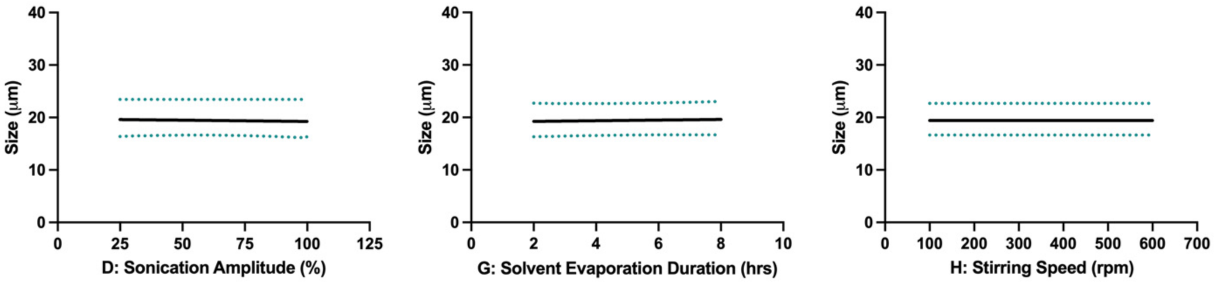

Figure S2. Single factor plots demonstrating the relationship between microparticle size and insignificant critical processing parameters, including (D) sonication amplitude (%) (n = 200), (G) solvent evaporation duration (n = 200), and (H) stirring speed (n = 200). The black solid line represents the expected trend, and the blue dotted line illustrates the confidence bands for that trend, as predicted by the RSM.

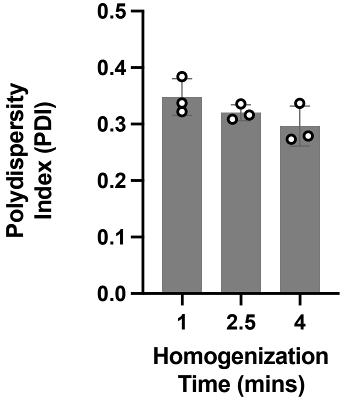

Figure S3. Microparticle polydispersity index (PDI) decreases slightly as homogenization time increases.

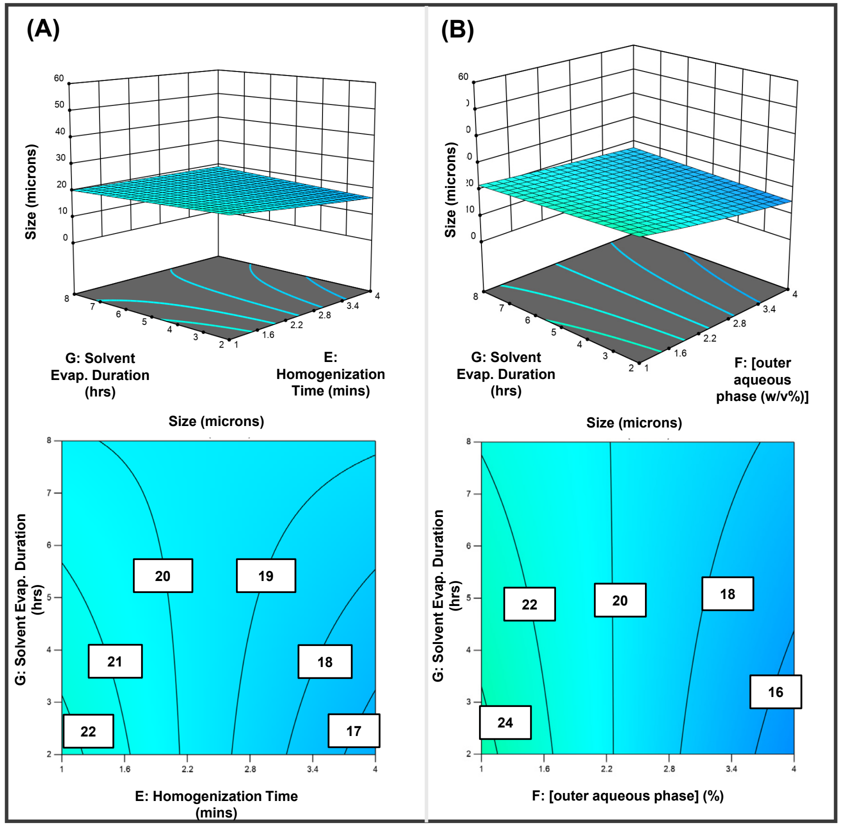

Figure S4. Homogenization time and outer aqueous phase concentration both interact with solvent evaporation duration to impact microparticle size. 3D surface and contour plots for the interactions of (A) homogenization time (mins) and solvent evaporation duration (hrs) and (B) [outer aqueous phase (w/v%)] and solvent evaporation duration (hrs). These interaction effects were found to be significant ($p < 0.5$).

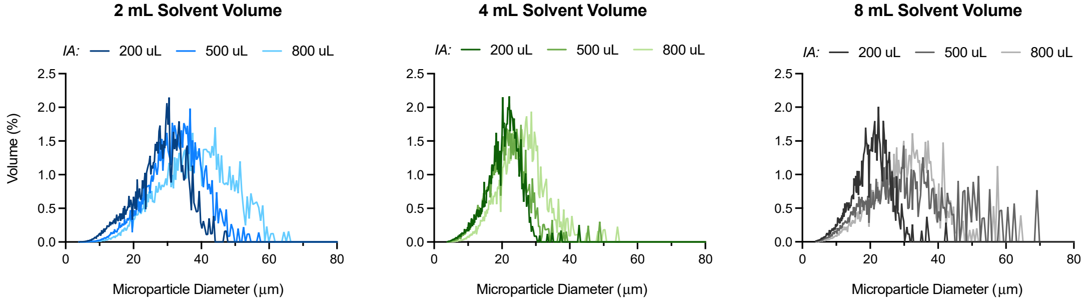

Figure S5. Increasing inner aqueous phase volume increases size and polydispersity of rhCCL22-MPs. Volume-weighted size distributions of rhCCL22-MPs formulated with various inner aqueous phase volumes (200, 500, 800 uL) at each solvent volume (2, 4, 8 mL), as determined by volume impedance measurements of n = 10,000 particles.

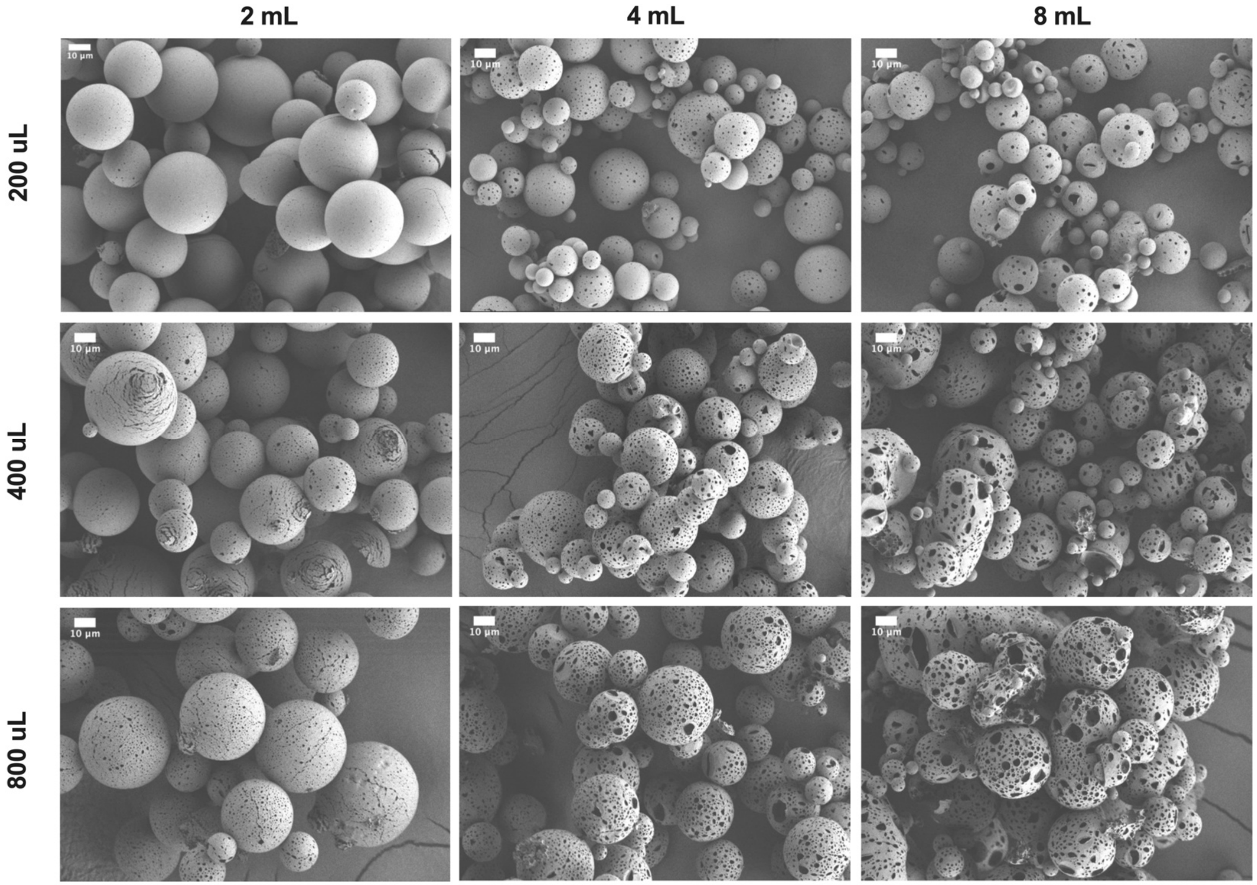

Figure S6. Microparticle surface porosity increases as solvent volume (mL) and inner aqueous phase volume (uL) increase. Scanning electron microscopy (SEM) images taken at 650x magnification. Scale bars correspond to 10 µm.

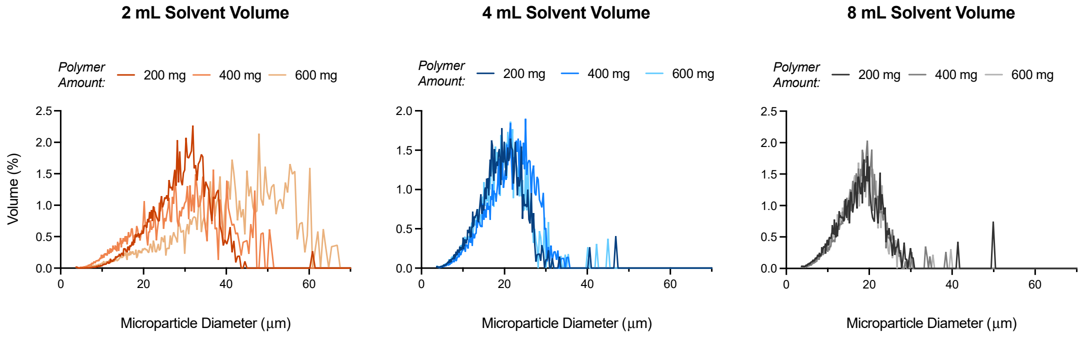

Figure S7. Increasing polymer amount at 2 mL of solvent volume impacts size and polydispersity of rhCCL22-MPs. Volume-weighted size distributions of rhCCL22-MPs formulated with various polymer amounts (200, 400, 600 mg) at each solvent volume (2, 4, 8 mL), as determined by volume impedance measurements of n = 10,000 particles.

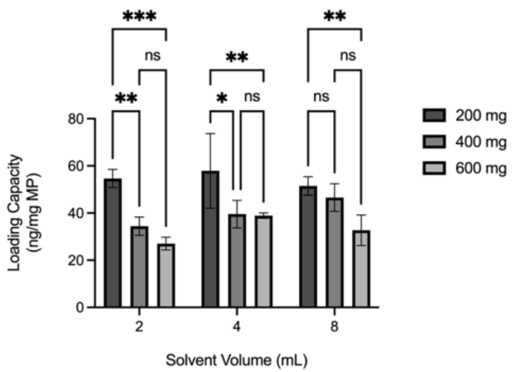

Figure S8. Impact of polymer amount and solvent volume on rhCCL22 loading capacity. Increasing polymer amounts within solvent volumes decreases loading capacity of rhCCL22-MPs (n = 3). * $p < 0.05$, ** $p < 0.01$, *** $p < 0.001$, ns indicates non-significant difference.
